# The impact of methamphetamine on psychosocial variables in patients from Iraq

**DOI:** 10.3389/fpsyt.2024.1376636

**Published:** 2024-05-08

**Authors:** Marwah Abbass, Nesif Al-Hemiary, Hayder B. Sahib

**Affiliations:** Iraqi Board for Medical Specialities, Baghdad, Iraq

**Keywords:** methamphetamine, crystal, psychosocial variables, urinary methamphetamine concentration levels (ng/ml), Baghdad

## Abstract

**Background:**

The current work reviews the psychosocial factors associated with different urinary methamphetamine concentration levels.

**Methods:**

From April to November 2023, 243 participants from Baghdad’s Al-Ataa Hospital were the subjects of a cross-sectional descriptive analysis study. We included 73 patients in this study.

**Result:**

The urinary methamphetamine concentration levels were from 3 to 92,274 ng/ml, with a mean ± SD of 10,873.6 ± 18,641. Patients diagnosed with major depression disorder exhibited higher scores on GHQ-30, UCLA, MOAS, and BDI-II with a significant *P*-value of 0.0001, 0.001, 0.0001, and 0.0001, consequently with an effect size of 0.015, 0.001, 1.05, and 3.24, respectively.

**Conclusions:**

The multi-screening test can produce a false positive. It frequently interferes with other drugs, especially antidepressants. This will result in patients being stigmatized and accused. On the other hand, those who accidentally come into contact with crystal smoke will experience the same withdrawal symptoms as the addicted patients. Their urinary methamphetamine level (titer) could have negative results. Urinary methamphetamine levels should be zero in healthy patients. In this situation, screening tests, expert opinion, and urine methamphetamine testing are strongly recommended.

## 1 Background

Methamphetamine is prescribed for obesity, narcolepsy, and attention deficit hyperactivity disorder (1). Methamphetamine is a member of a group of synthetic compounds referred to as amphetamines (2). Amphetamines are referred to as ice and crystal meth on the street. Shabu was its Egyptian name (3). There are four commonly used amphetamines in the US, and methamphetamine is one of them (4). In Iraq, dextroamphetamine is in addition to methamphetamine (5). People who utilize methamphetamine frequently take polydrugs, which needs to be evaluated. Higher rates of dependency and hazardous use are linked to crystalline methamphetamine (6). The primary effects are neurocognitive; these include depression, psychosis, and other neuropsychiatric and cognitive consequences, in addition to a pleasurable and energetic mood (7). Chronic methamphetamine usage causes neurotoxicity in dopaminergic axon terminals, which is linked to decreased dopamine synthesis and decreased expression of the dopamine transporter despite the fact that this causes acute elevations in dopamine and norepinephrine signaling that drive the methamphetamine-associated euphoria (8). One of the key contributing factors to the development and maintenance of drug misuse is distress tolerance (9). When a substance is used repeatedly, tolerance and dependent use develop over weeks or months. A disorder of the regulation of stimulant use, stimulant dependence is caused by the repeated or continuous use of stimulants and is characterized by subjective cravings and strong internal desires to use (i.e., impaired control over use, prioritizing use over other activities, and persistent use despite harm or negative consequences). When stimulants are used consistently for at least a month—that is, nearly every day—for at least a year, signs of dependence typically become apparent (6). Stimulant use disorder is defined by a pattern of usage that results in clinically significant impairment or distress, escalation of the dose used, unsuccessful attempts to reduce, cravings, problems in social or professional settings, and physical or psychological issues (6).

### 1.1 Rationale of the study

Al-Hemiary et al. (2014) in Iraq verified a sharp rise in the use of illicit Amphetamine-Type Stimulants, especially methamphetamine, and captagon. Also, Al-Basrah governorate showed a particularly noticeable increase (10, 11). Ciccarone and Shoptaw (2022) examined an alarming shift in the global overdose mortality rate from opioids, from the third wave to a more severe fourth wave, characterized by methamphetamine and cocaine usage (12). The psychological strains and extreme stress that members of society have experienced as a result of terrorism and the precarious social and economic circumstances that Iraq is currently experiencing are among the reasons why drug usage has become more common in recent years. There is proof that drug outbreaks result in major issues (13).

Abuse of methamphetamine results in psychological symptoms, such as depression, and irreparable brain damage (14). Subjectively upsetting, loneliness is a feeling that develops when someone feels that they are not interacting with others enough, either in terms of quantity or quality. Several researches have linked addictive behaviors to mental health issues like anxiety, depression, and loneliness. More people feel lonely when they abuse drugs than when they do not. Drug misuse and participation in dangerous activities are more likely to occur in lonely people (15).

Whether or not it is thought to be caused by methamphetamine, a diagnosable psychotic disease must have symptoms that go beyond what would be expected from intoxication or withdrawal from methamphetamine. When psychotic symptoms that began in the context of methamphetamine use persist for more than a month after stopping methamphetamine use or when symptoms resurface in the absence of methamphetamine use, differential diagnostic problems regarding the etiology of a psychotic condition among methamphetamine users generally arise (16).

According to L. Curran et al., three categories of patient diseases could be the focus of prevention and therapy, and mental health disorders constitute one of them. Several risk markers were found to indicate that methamphetamine users were younger and more likely to be male than the total hospitalized population under investigation. Users of methamphetamine were significantly more likely to have anxiety and depression than nonusers, and they were also more likely to have a lower socioeconomic status (17).

SA Nia et al. claim that while methamphetamine use increased rapidly, so did the drug’s supply in Iran. Methamphetamine holds the leading position in Iran’s pharmaceutical industry (18).

### 1.2 Aim of the study

This study aims to investigate the relationship between methamphetamine urine concentration levels and psychosocial variable parameters in patients with substance addiction or abuse.

### 1.3 Study question

Do urinary methamphetamine concentration levels have a significant association with psychological and behavioral characteristics, such as depression, loneliness, and aggression, in patients with substance addiction or abuse?

### 1.4 Study hypothesis

Patients with methamphetamine urine concentrations above 300 ng/mL do not demonstrate statistically significant associations with non-inferior levels of psychosocial variable parameters, including loneliness, social isolation, and depression, in comparison to aggressive behaviors.

## 2 Materials and methods

### 2.1 Research ethics

The current study has received ethical approval from (1) the Ethical Committee, Iraqi Board for Medical Specializations. The approval was granted according to number 739 on the 27th of February 2023; (2) the Iraqi Ministry of health. The approval was granted according to protocol number 2 in 2021 (https://moh.gov.iq/?page=51); and (3) Al-Rusafa health directorate, Baghdad. The approval was granted according to number 42397 on the 20th of March 2023. Additionally, this study is a research output of the Iraqi Medical Board thesis, clinical pharmacy department.

### 2.2 Study structure

A cross-sectional descriptive analysis study would be conducted on 243 participants (all patients were male) from Al-Ataa Hospital in Baghdad, Iraq, between April 2023 and November 2023. The patients were seeking treatment for methamphetamine dependence. The flowchart describes the work plan in **Appendix A**.

### 2.3 Study participants

**Figure 1:**
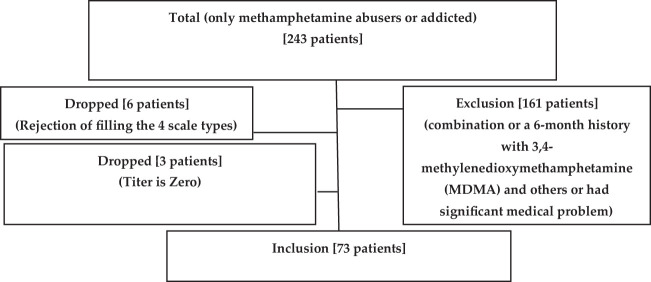
Inclusion flow chart.

In this study, out of 161 patients, 73 patients were admitted due to methamphetamine contaminants (combination, a 6-month history with multi-drugs including 3,4-methylenedioxymethamphetamine or a significant medical problem). Nine patients were dropped from the study (six patients because of rejection filling out the four scale types and three patients due to their pure titer result being zero) as shown in **Figure 1**.

#### 2.3.1 Inclusion criteria

1. Patients eligible for the study must have a history of methamphetamine addiction or abuse.
2. They must have undergone a multi-drug screening test.
3. Patients may fall into one of the following categories:

- Newly admitted patients with methamphetamine use only.

- Routinely admitted patients with methamphetamine use only.

- Routinely admitted patients with a history of multidrug abuse, provided that they have abstained from all drugs for at least 6 months except methamphetamine.

4. Methamphetamine urine concentration must be tested, with patients falling into one of two categories:

- Positive for detectable methamphetamine urine concentration.

- Negative, indicating either past methamphetamine use or cessation of use.

#### 2.3.2 Exclusion criteria

1- Participants who had abused drugs other than methamphetamine for at least 6 months, except amphetamine.
2- Patients with a previous history of mental or significant medical illness.
3- Patients who fulfilled the Diagnostic and Statistical Manual of Mental Disorders-5 criteria for dependency on any compounds other than methamphetamine and those who show positive for many drugs during drug screening.

### 2.4 Study variables

Using the survey, data collection encompassed several key variables representing different aspects of the study objectives: (1) socio-demographic characteristics of the studied population: This field included information about participants’ age, gender, educational background, marital status, and employment status; (2) patterns of methamphetamine abuse associated with different methamphetamine urine concentration levels: In this part, the collected data from the condition of receiving methamphetamine, duration of discontinuation, and years of abusing and routine reviewing; (3) psychosocial variables associated with different methamphetamine urine concentration levels: This is the major part of assessing patients by using the tools: General Health Questionnaire-30, Beck Depression Inventory-II, University of California, Los Angeles Loneliness Scale, and Modified Overt Aggression Scale; (4) comparison of laboratory data with methamphetamine urine concentration levels: to mainly assess the liver and renal function; and (5) medication decision with methamphetamine urine concentration levels: this section deals with the drugs that are used to relieve the symptomatic effect of methamphetamine, which include antipsychotics, antidepressants, analgesia, sedatives, anticonvulsants, and GABA analog (antiseizure). By analyzing these variables, the study aimed (1) to determine the psychosocial variables associated with different urinary methamphetamine concentration levels and (2) to find the relationship between different psychosocial variables and other parameters with urinary methamphetamine concentration levels.

### 2.5 Criteria for eligibility

Diagnosis of methamphetamine use disorder was done by the initial urine screening test, the stated history of misuse, the Diagnostic and Statistical Manual of Mental Disorders (DSM) -5, and major depression disorder (MDD).

(1) Urinary drug screening by multi-drug rapid test panel, (2) amphetamines II (AMPS2) urinary test from Roche/Hitachi company. A Cobas C analyzer is used. One single AMPS2 test costs US $9. In our work, some patients used three tests due to the turbidity of urine, (3) General Health questionnaire GHQ30 (19) (with permission from a publishing company Mapi Research Trust with order number 2315915), (4) Beck Depression Inventory-II (20): the Arabic version was used, (5) University of California, Los Angeles (version 3) (15): the Arabic version was used (21), and (6) Modified Overt Aggression Scale: the Arabic version was used (3, 22, 23).

### 2.6 Data analysis

(1) Sample size determination: 73 patients.
(2) The statistical tests used: chi-square, mean with or without SD, ***P***-value, and ***t***-test with Cohen’s ***d*** value.
(3) The statistical package used is IBM SPSS version 29.

## 3 Results

The number of patients included in the current study is 73. The studied sample was analyzed across two groups: group B (the control group) representing individuals with normal mental health (comprising normal, mild, and borderline cases) and group A (the major group) representing individuals diagnosed with major depressive disorder (MMD) encompassing moderate, severe, and extreme depression. 

The socio-demographic features shown in **Table 1** illustrate that all patients are male. No statistically significant associations were found between the two groups across all variables (*p* > 0.05 for all comparisons), including educational level (*p* = 0.686), occupation (*p* = 0.863), residence (*p* = 0.839), financial status (*p* = 0.558), and marital status (*p* = 0.897). While age exhibited variation in major depressive disorder (MDD) prevalence across different age groups, the overall difference was not statistically significant (*p* = 0.349).

**Table 1:** Socio-demographic features of the studied sample.

		Group B—normal (normal + mild + borderline) (1-20) *n* = 23		Group A—major depression disorder (MMD) (moderate + severe + extreme depression) (≥21) *n* = 50		P value
		No.	%	No.	%	
Age (years)	12–19	6	26.1	7	14.0	0.349
	20–29	11	47.8	32	64.0	
	30–37	6	26.1	11	22.0	
Gender	Male	23	100.0	50	100.0	–
	Female	–	–	–	–	
Educational level	Illiterate	8	34.8	14	28.0	0.686
	Primary/secondary	14	60.9	35	70.0	
	College/higher	1	4.3	1	2.0	
Occupation	Unemployed	13	56.5	25	50.0	0.863
	Employed	1	4.3	3	6.0	
	Worker	9	39.1	22	44.0	
	Student	–	–	–	–	
Residence	Baghdad center	14	60.9	30	60.0	0.839
	Baghdad periphery	3	13.0	9	18.0	
	Others	6	26.1	11	22.0	
Financial status	Low	15	65.2	29	58.0	0.558
	Middle	8	34.8	21	42.0	
	High	–	–	–	–	
Marital status	Single	15	65.2	30	60.0	0.897
	Married	7	30.4	18	36.0	
	Widowed	–	–	–	–	
	Divorced	1	4.3	2	4.0	

Significant difference between percentages using Pearson chi-square test (χ^2^ test) at 0.05 level.

Patterns of Methamphetamine abuse: **Table 2** illustrates significant associations that emerge between various clinical factors and methamphetamine abuse patterns. Patients diagnosed with major depressive disorder (MDD) exhibited notably higher urine methamphetamine concentrations (*p* = 0.001). Admission status also played a crucial role, with newly admitted and routinely admitted patients displaying significantly elevated levels (*p* = 0.011 and *p* = 0.009, respectively). Moreover, longer periods of methamphetamine discontinuation were linked with lower concentrations (*p* = 0.021, *p* = 0.020, *p* = 0.008 for 14–20 days, 21–27 days, and 28 days or more, respectively), while longer durations of methamphetamine use correlated with higher concentrations (*p* = 0.015 and *p* = 0.029 for 1–4 years and 5–8 years). Routine reviews every 3 months were associated with lower concentrations (*p* = 0.009), and individuals testing positive for methamphetamine had significantly higher concentrations (*p* = 0.044).

**Table 2:** Patterns of methamphetamine abuse associated with different methamphetamine urine concentration levels.

		Methamphetamine concentration level in urine (ng/mL)				*P* value	Effect size
		Normal (normal + mild+ borderline) (1–20) (*n* = 23)		Group A—major depression disorder (MMD) (moderate + severe + extreme depression) (21–50) (*n* = 50)			
		No.	Mean ± SD	No.	Mean ± SD		
Patient is receiving methamphetamine	Yes	23	275.30 ± 700.55	50	15,744.44 ± 20,820.92	0.001^a^	2.53^b^
	No	–	–	–	–	–
Conditions for receiving methamphetamine	Newly admitted	13	434.54 ± 913.79	35	17,925.23 ± 23,524.17	0.011^a^	1.23^b^
	Routinely admitted with MA	10	68.30 ± 45.70	15	10,655.93 ± 11,563.55	0.009^a^	3.23^b^
	Routinely admitted with MA and other drugs	–	–	–	–	–	
Duration of methamphetamine discontinuation (days)	0–3	3	1,429.33 ± 1,746.5	28	21,870.71 ± 22,725.21	0.136	
	4–6	–	–	5	7,868.80 ± 7,947.24	–	
	7–13	3	188.00 ± 97.53	9	11,931.11 ± 23,148.45	0.415	
	14–20	3	37.33 ± 28.02	2	7,304.50 ± 3,092.18	0.021^a^	3.03^b^
	21–27	2	66.00 ± 89.10	1	3,502.00 ±	0.020^a^	1.88^b^
	≥28 days	12	103.00 ± 57.04	5	2,001.40 ± 2,274.19	0.008^a^	3.54^b^
Duration of taking methamphetamine (years)	<1 year	3	1,179.33 ± 1,912 ± .4	9	19,014.78 ± 22,125.00	0.207
	1–4	14	102.29 ± 57.73	32	15,543.69 ± 22,616.54	0.015^a^	2.71^b^
	5–8 years	6	227.00 ± 332.87	9	13,187.89 ± 12,810.07	0.029^a^	2.04^b^
Undergone routine review (every 3 months)	Yes	10	68.30 ± 45.70	15	10,655.93 ± 11,563.55	0.009^a^	2.14^b^
	No	13	434.54 ± 913.79	35	17,925.23 ± 23,524.17	0.011^a^	
Methamphetamine concentration level in urine	Positive	2	2,130.00 ± 1,776.3	45	17,473.56 ± 21,264.86	0.318	
	Negative	21	98.67 ± 73.11	5	182.40 ± 104.18	0.044^a^	0.60^b^

a Significant difference between two independent means using Student’s *t*-test at 0.05 level.

b Effect size was measured using Cohen’s *d* value.

**Figure 2:**
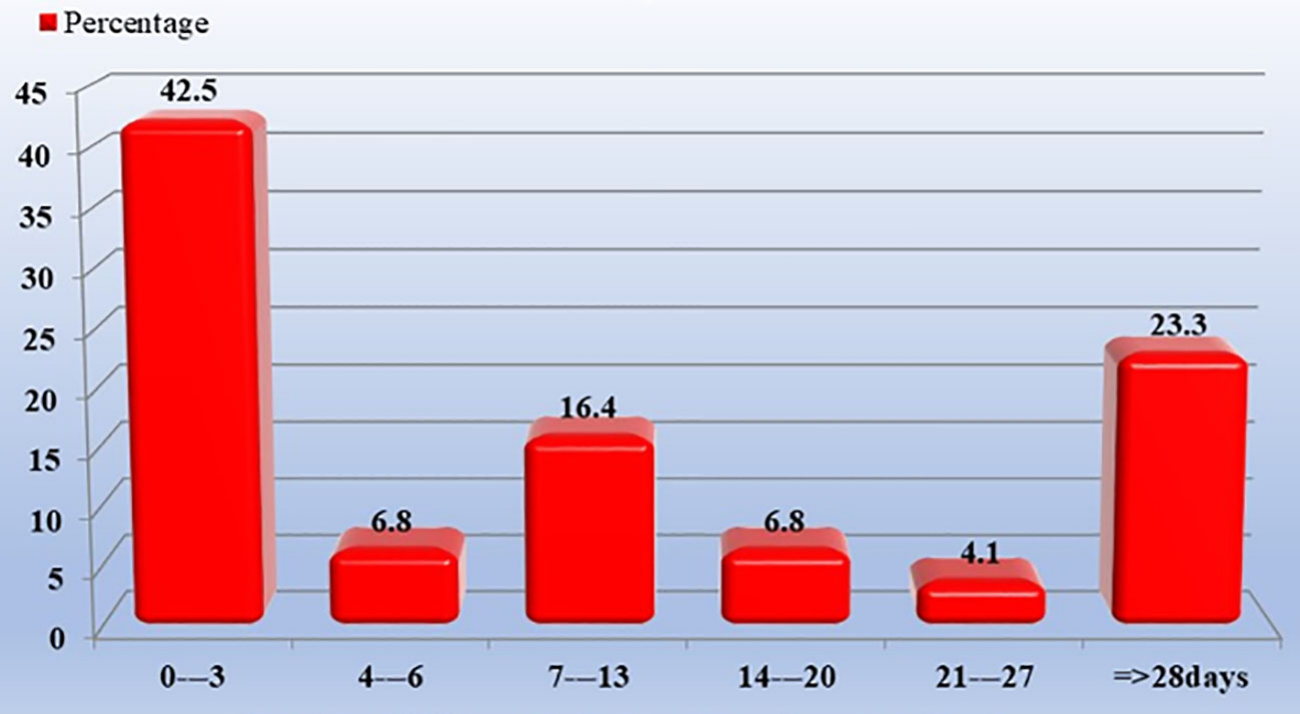
Duration of methamphetamine discontinuation (days).

**Figure 2** shows the duration of methamphetamine discontinuation (days). A duration from 0 to 3 day(s) had 42.5% and that of 28 days or more had 23.3%.

Methamphetamine concentration levels were determined using the Amphetamines II (AMPS2) test. In the sample (73 patients), the methamphetamine concentration levels in urine were from 3 to 92,274 ng/mL, with mean ± SD 10,873.6 ± 18,641.

Psychosocial Variables assessment: **Table 3** depicts significant associations with notable effect sizes. Patients diagnosed with major depression disorder (MDD) exhibited higher scores on the General Health Questionnaire (GHQ-30) compared to those without MDD (*p* = 0.0001, Cohen’s *d* = 0.015). Additionally, higher urine methamphetamine concentrations were associated with increased loneliness, as measured by the University of California, Los Angeles (UCLA) Loneliness Scale (*p* = 0.001, Cohen’s *d* = 0.001). Levels of aggression, assessed using the Modified Overt Aggression Scale (MOAS), were significantly elevated in individuals with higher methamphetamine concentrations (*p* = 0.0001, Cohen’s *d* = 1.05). Beck Depression Inventory (BDI-II) scores demonstrated a significant increase with higher methamphetamine concentrations, indicating more severe depression (*p* = 0.0001, Cohen’s *d* = 3.24). Patients ranging from moderate to extreme depression levels meet the DSM-5 criteria for MDD.

**Table 3:** Psychosocial variables associated with different methamphetamine urine concentration levels.

		Methamphetamine concentration level in urine (ng/mL)				*P* value	Effect size
		Group B—Normal (normal+ mild+ borderline) (1–20) *n* = 23		Group A—major depression disorder (MMD) (moderate + severe + extreme depression) (21–50) *n* = 50			
		No.	Mean ± SD	No.	Mean ± SD		
General Health Questionnaire (GHQ-30)	Mean ± SD	39.48 ± 10.26		51.06 ± 11.99		0.0001^a^	0.015^b^
	No significant distress (0–9)	–	–	–	–	–	
	Mild (10–19)	–	–	–	–	–	
	Moderate (20–29)	3	46.67 ± 22.23	–	–	–	
	Severe (30–90)	20	309.60 ± 747.47	50	15,744.44 ± 20,820.92	0.002^a^	0.753^b^
ULCA Loneliness Scale	Mean ± SD	46.30 ± 8.15		49.28 ± 6.12		0.087	0.001^b^
	Low (20–34)	4	71.00 ± 38.67	–	–	–	
	Moderate (35–49)	9	107.67 ± 39.40	23	2,389.09 ± 2,631.18	0.015^a^	0.901^b^
	High loneliness (50–60)	10	507.90 ± 1,044.52	27	27,121.22 ± 22,780.44	0.001^a^	1.186^b^
Modified Overt Aggression Scale (MOAS)	Mean ± SD	1.48 ± 1.93		4.84 ± 3.30		0.0001^a^	1.05^b^
	Absence of aggression behavior (0)	4	868.25 ± 1,678.51	3	409.00 ± 113.66	0.664	
	Minimal (little to no) (1–5)	18	153.44 ± 193.93	32	10,523.22 ± 14,912.25	0.005^a^	0.706^b^
	Mild (occasional mild) (6–10)	1	97.00 ±	9	25,394.78 ± 23,453.53	0.336	
	Moderate (frequent/more intense) (11–15)	–	–	6	36,783.17 ± 31,228.19	–	
	Severe (frequent and intense) (16–20)	–	–	–	–	–	
Beck Depression Inventory (DBI-II) Level of Depression	Mean ± SD	11.48 ± 4.09		40.48 ± 8.94		0.0001^a^	3.24^b^
	Normal ups and downs (1–10)	11	158.18 ± 250.89	–	–	–	
	Mild mood disturbance (11–16)	9	112.00 ± 64.23	–	–	–	
	Borderline clinical depression (17–20)	3	1,194.67 ± 1,898.4	–	–	–	
	Moderate (21–30)	–	–	8	6,147.63 ± 13,765.49	–	
	Severe (31–40)	–	–	21	6,385.62 ± 7,116.87	–	
	Extreme depression (>40)	–	–	21	28,759.19 ± 25,267.97	–	

a Significant difference between two independent means using Student’s *t*-test at 0.05 level.

b Effect size was measured using Cohen’s *d* value.

**Figure 3:**
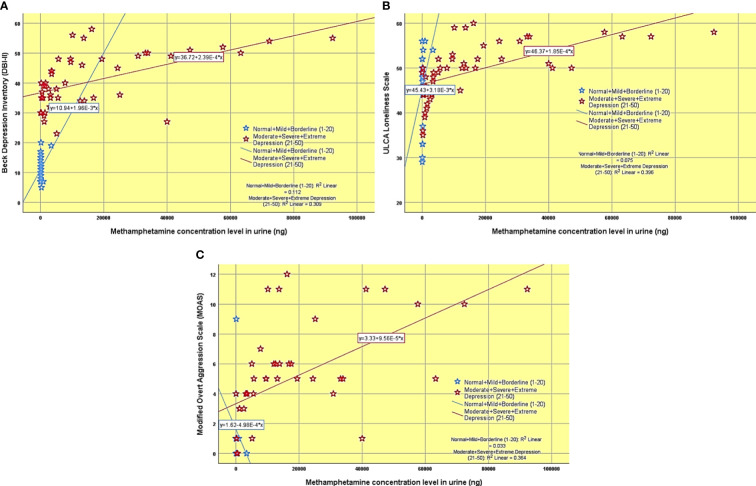
**(A)** The Beck Depression Inventory-II score differs between group A (MDD) and group B (normal) in association with varying methamphetamine urine concentration levels. **(B)** University of California, Los Angeles (UCLA) scale differs between group A (MDD) and group B (normal) in association with varying methamphetamine urine concentration levels. **(C)** Modified Overt Aggression (MOAS) scale differs between group A (MDD) and group B (normal) in association with varying methamphetamine urine concentration levels.

**Figure 3A** shows the Beck Depression Inventory-II across group A (MDD) and group B (normal) associated with different methamphetamine urine concentration levels.

**Figure 3B** shows the University of California, Los Angeles (UCLA) scale across group A (MDD) and group B (normal) associated with different methamphetamine urine concentration levels.

**Figure 3C** shows the Modified Overt Aggression (MOAS) scale across group A (MDD) and group B (normal) associated with different methamphetamine urine concentration levels.

Laboratory data with methamphetamine: **Table 4** shows the laboratory data with methamphetamine. Significant differences were observed in creatinine levels (*p* = 0.0001, Cohen’s *d* = 1.74), hemoglobin (Hb) levels (*p* = 0.031, Cohen’s *d* = 0.46), and aspartate aminotransferase (SGOT) levels (*p* = 0.031, Cohen’s *d* = 1.18) between patients with normal and elevated methamphetamine concentrations.

**Table 4:** Comparison of laboratory data with urinary methamphetamine concentration levels.

	Group B—normal (normal + mild + borderline) (1–20) (*n* = 23)	Group A—major depression disorder (MMD) (moderate + severe + extreme depression) (≥21) (*n* = 50)	*P* value	Effect size
	Mean ± SD	Mean ± SD		
Blood urea	29.11 ± 6.30	27.34 ± 5.73	0.240	
Creatinine	0.63 ± 0.31	0.85 ± 0.15	0.0001^a^	1.74^b^
WBC	7.47 ± 2.13	7.73 ± 2.18	0.626	
Hb	14.24 ± 1.47	14.87 ± 0.92	0.031^a^	0.46^b^
Alanine aminotransferase (SGPT)	29.96 ± 16.36	36.38 ± 17.66	0.144	
Aspartate aminotransferase (SGOT)	25.90 ± 11.91	33.38 ± 14.11	0.031^a^	1.18^b^

a Significant difference between two independent means using Student’s *t*-test at 0.05 level.

b Effect size was measured using Cohen’s *d* value.

Significant differences were found in medication decisions (as detox medications), as shown in **Table 5**, associated with methamphetamine urine concentration levels. Olanzapine usage was significantly higher in patients with elevated methamphetamine concentrations (*p* = 0.004, Cohen’s *d* = 1.079). Fluoxetine (*p* = 0.005, Cohen’s *d* = 1.046) and analgesia use without specific medication (*p* = 0.001, Cohen’s *d* = 1.074) were also notably higher in this group. Furthermore, sedative use, specifically benzodiazepine (*p* = 0.001, Cohen’s *d* = 1.446), and anticonvulsant use, particularly carbamazepine (*p* = 0.003, Cohen’s *d* = 1.041), demonstrated significant differences between the two groups.

**Table 5:** Medication decision with methamphetamine urine concentration levels.

		Methamphetamine concentration level in urine (ng/mL)				*P* value	Effect size
		Group B—normal (normal + mild + borderline) (1–20) *n* = 23		Group A—major depression disorder (MMD) (moderate + severe + extreme depression) (21–50) *n* = 50			
		No.	Mean ± SD	No.	Mean ± SSD		
Antipsychotics	Olanzapine	16	97.19 ± 58.21	43	15,530.98 ± 20,301.03	0.004^a^	1.079^b^
	Resperidone	2	168.50 ± 184.55	4	25,091.50 ± 32,294.39	0.362	
	No	5	888.00 ± 1,440.89	3	6,341.33 ± 9,669.88	0.238	
Antidepressants	Fluoxetine	16	108.56 ± 76.95	39	16,539.74 ± 22,201.23	0.005^a^	1.046^b^
	Escitalopram	4	74.25 ± 59.03	9	15,543.56 ± 16,048.38	0.087	
	No	3	1,432.67 ± 1,742.5	2	1,140.00 ± 45.25	0.836	
Analgesia	Chlodiazepoxide (Libirium)	3	175.33 ± 108.08	5	8,776.40 ± 9,335.52	0.173	
	No	20	290.30 ± 751.81	45	16,518.67 ± 21,649.93	0.001^a^	1.074^b^
Sedatives	Benzodiazepine	3	1,186.67 ± 1,905.1	1	416.00 ±	0.760	
	No	20	138.60 ± 188.14	49	16,057.27 ± 20,917.63	0.001^a^	1.446^b^
Anticonvulsants	Carbamazepine (Tegretol)	17	99.18 ± 56.54	36	17,624.67 ± 23,347.82	0.003^a^	1.041^b^
	Sod. Valproate (Depakine)	–	–	5	8,803.00 ± 10,559.57	–	
	No	6	774.33 ± 1,320.93	9	12,079.89 ± 12,388.11	0.047^a^	1.327^b^
GABA analog (antiseizure)	Gabapentin	6	73.83 ± 48.72	20	14,313.55 ± 17,379.71	0.059	
	No	17	346.41 ± 808.38	30	16,698.37 ± 23,070.16	0.006^a^	0.996^b^

a Significant difference between two independent means using Student’s *t*-test at 0.05 level.

b Effect size was measured using Cohen’s *d* value.

In **Table 2**–**5**, the *p*-values were computed at the 0.05 level using Student’s *t*-test with Cohen’s *d* value. According to Cohen’s guidelines, the effect size can be interpreted as small (around 0.2), medium (around 0.5), or large (around 0.8).

## 4 Discussion

The occurrence of major depression disorder (MMD) and its association with methamphetamine abuse was highlighted, emphasizing the severity of mental health comorbidities in methamphetamine users and echoing observations by Hashisha et al. (2022) (3). The findings underscore the profound impact of methamphetamine on psychosocial factors, particularly among individuals with varying urine concentration levels of the substance.

The observed gender disparity in rehabilitation facilities, attributed to male-exclusive wards, underscores the need for gender-sensitive treatment approaches. Moreover, the socio-demographic profile of the studied sample, with a prevalence of urban residency and primary to secondary education attainment, resonates with the findings of Al-imam et al. (2023) and Chachan et al. (2022), suggesting a correlation between drug availability and population density (4, 5). However, the discrepancy in illiteracy rates, covering one-third of the sample, deviates from earlier studies, indicating potential unique local factors or variations in educational infrastructure (4, 5). Similarly, marital status trends align with those observed by Chachan et al., suggesting societal or cultural influences on divorce rates (5).

The findings align with global perspectives linking unemployment among young individuals with increased drug consumption, as highlighted in previous research (3, 24). Another study from Erbil declares the comparable consequences published by N. Mahmood et al. (25). Moreover, the significant rise in methamphetamine screening results corroborates data from Curran et al. (2022), emphasizing the widespread prevalence of substance abuse within society (17). Notably, minor cross-reactivity in multidrug screening tests may occur, potentially resulting in false positive amphetamine screening results (20).

Exclusion criteria ensuring the focus on individuals with methamphetamine addiction or abuse without pre-existing mental or medical conditions align with the study’s objective. The prolonged duration of methamphetamine abuse observed in the study cohort raises clinical concerns regarding the chronicity of substance use disorders and the challenges associated with long-term management and intervention, consistent with previous research (13).

Assessments of psychosocial variables reveal severe distress levels among the sample, consistent with prior findings (26) and echoing observations by Hashisha et al. (2022) (3). Moreover, correlations between methamphetamine abuse and scores on psychological scales highlight the global impact of substance abuse on mental wellbeing (15).

The psychological impact of drug addiction, particularly methamphetamine use, extends beyond mere substance dependence, encompassing a spectrum of social and psychological challenges. This study echoes prior research (27). The concerning relationship between methamphetamine concentration levels and psychological distress underscores the heightened vulnerability of individuals with methamphetamine addiction to mood disturbances, emphasizing the importance of comprehensive psychiatric assessment and tailored interventions (28). Additionally, associations between methamphetamine use and aggression align with previous research, emphasizing the need for vigilant monitoring and targeted interventions to mitigate aggression (3, 28).

The utilization of specific medications such as olanzapine, fluoxetine, and carbamazepine in managing methamphetamine addiction emphasizes the diverse treatment approaches influenced by physician preferences and patient responses, consistent with prior research (6, 29).

The prevalence of major depression disorder (MMD) within the cohort of individuals grappling with methamphetamine abuse unveils a striking correlation that echoes global observations. This connection highlights a profound vulnerability to mood disturbances among this demographic group (28). Delving deeper, the study sheds light on how varying levels of methamphetamine concentration in urine intertwine with psychological distress, magnifying the substantial toll methamphetamine abuse exacts on mental wellbeing (28). These revelations not only emphasize the urgency of acknowledging and addressing the intricate relationship between substance abuse and mental health but also underline the need for nuanced interventions tailored to this complexity.

What is particularly intriguing is how these findings resonate with prior research by Hashemzadeh et al. (2021) and Marquez-Arrico et al. (2020), where similar patterns were observed (30, 31). This confluence of evidence adds weight to the argument that major depression disorder (MMD) is not just a concurrent issue but rather an integral facet of the intricate tapestry of challenges faced by those entangled in methamphetamine abuse.

### 4.1 Limitation and strength of the study

The study’s sample size is small, yet it comprises individuals exclusively using pure methamphetamine, which is typically challenging to encounter. The presence of other mental disorders or significant medical illnesses is another factor. These individuals were sourced from a population known to consume raw or combination drugs. Variations in urinary multi-drug test thresholds across centers necessitate remaining within a single facility. Furthermore, the study’s focus on detoxification programs for long-term drug abusers represents another potential limitation. Moreover, the study’s cross-sectional design limits its ability to establish causality or assess long-term effects. Despite these limitations, the study provides valuable insights into the complex relationship between methamphetamine use and various psychosocial factors, highlighting the need for further research in this area.

## 5 Conclusions

Concentrations above 300 ng/mL, which is the detectable level, demonstrate statistically significant non-inferior levels of psychosocial variables, including loneliness, social isolation, and depression, in comparison to aggressiveness behaviors. Methamphetamine urinary concentration levels increase with a dramatically significant elevation of depression, loneliness, and social isolation and a mild significant elevation of aggressiveness behavior. The multi-screening test could give a false positive. It tends to interact with other medications, particularly antidepressants. This will lead to accused patients and stick a stigma on them—a negative result with a patient who does not have any previous diseases and/or problems but undergoes withdrawal symptoms of drug abuse. Methamphetamine smoking makes people become abusers accidentally. We highly recommended using a urinary methamphetamine test in addition to a screening test and specialist decision.

## Data availability statement

The raw data supporting the conclusions of this article will be made available by the authors, without undue reservation.

## Ethics statement

The studies involving humans were approved by Research Ethics: The current study has received ethical approval from: The Ethical Committee, Iraqi Board for Medical Specializations. The approval was granted according to number 739 on the 27th of February 2023, The Iraqi Ministry of Health. The approval was granted according to protocol number 2 in 2021, Al-Rusafa Health Directorate, Baghdad. The approval was granted according to number 42397 on the 20th of March 2023. Additionally, this study is a research output of the Iraqi Medical Board thesis, Clinical Pharmacy Department. The studies were conducted in accordance with the local legislation and institutional requirements. Written informed consent for participation in this study was provided by the participants’ legal guardians/next of kin.

## Author contributions

MA: Conceptualization, Data curation, Formal Analysis, Funding acquisition, Investigation, Methodology, Project administration, Resources, Software, Supervision, Validation, Visualization, Writing – original draft, Writing – review & editing. NA-H: Conceptualization, Data curation, Formal Analysis, Funding acquisition, Investigation, Methodology, Project administration, Resources, Software, Supervision, Validation, Visualization, Writing – review & editing. HS: Conceptualization, Data curation, Formal Analysis, Funding acquisition, Investigation, Methodology, Project administration, Resources, Software, Supervision, Validation, Visualization, Writing – review & editing.
